# The Effect of Education Based on Kolb's Learning Style on Selfcare Behaviors of the Elderly with Type II Diabetes: A Randomized, Clinical Trial

**DOI:** 10.4314/ejhs.v31i6.24

**Published:** 2021-11

**Authors:** Omolbanin Bagchegi, Mahbubeh Tabatabaeichehr, Hossein Lashkardoost, Hamed Mortazavi

**Affiliations:** 1 Student Research Committee, Department of Geriatric Nursing, Geriatric Care Research Center, School of Nursing and Midwifery, North Khorasan University of Medical Sciences, Bojnurd, Iran; 2 Geriatric Care Research Center, Department of Midwifery, School of Medicine, North Khorasan University of Medical Sciences, Bojnurd, Iran; 3 School of Public Health, North Khorasan University of Medical Sciences, Bojnurd, Iran; 4 Geriatric Care Research Center, Department of Geriatric Nursing, School of Nursing and Midwifery, North Khorasan University of Medical Sciences, Bojnurd, Iran

**Keywords:** Self-care, Education, Kolb's learning style, Elderly, Type II diabetes

## Abstract

**Background:**

Individualized patient education can help older people with diabetes to improve their self-care behaviors and effectively manage their disease. Identifying patients' preferred learning style can be a useful way to optimize learning opportunities. The aim of this study was to investigate the effect of education based on Kolb's learning style on selfcare behaviors of older people with type II diabetes.

**Methods:**

This randomized clinical trial was conducted on 62 older adults with type II diabetes referred to the diabetes center of Imam Reza Hospital in Bojnourd, Iran, from May to November 2020. Participants who met the inclusion criteria were randomly allocated into two groups of intervention (n=34) and control (n=28). Participants in intervention group received the educational intervention based on their learning style (described by Kolb as accommodating, diverging, converging and assimilating) in four 45 minutes-sessions (two sessions per week). In the control group, the same educational content was presented with a lecture and a booklet. Participants in both groups completed the self-care questionnaires before and one month after the intervention.

**Results:**

The mean self-care scores of the older adult patients with type II diabetes based on Kolb's learning style after the educational intervention in the intervention and control groups were 58.15 (SD=10.71) and 44.7 (SD=12.12), respectively (P<0.001). Accommodating Kolb's learning style-based education was most effective in improving self-care practice in older adult patients with type II diabetes (P<0.001).

**Conclusion:**

Education based on Kolb's learning style is effective in improving self-care behaviors of older people with type II diabetes. Therefore, it is suggested to consider Kolb's learning style in the educational process of older adults with type II diabetes to improve self-care practice in these patients.

## Introduction

Population aging is a major challenge for the healthcare system (1). It is estimated that the number of people over the age of 60 will nearly double by 2050 (2, 3). Also, it has been estimated that as much as 30% of Iran's population will be aged 60 and older, by 2050 (4, 5). On the other hand, diabetes is one of the age-related diseases that occur more frequently in the older people (6). The global diabetes prevalence has been reported as 19.3% and it affects almost 15% of Iranian older adult population (7, 8).

In diabetic patients, effective performance of self-care behaviors is incredibly important for disease control, prevention of long-term complications and improving patients' quality of life. Therefore, diabetes self-care education deserves adequate clinical attention (9, 10). Education has been considered as a critical component of effective diabetes care. It can help patients and healthcare providers to recognize and understand the health need of patients, changing health behaviors, improving self-care and decision-making process for patients with diabetes (11, 12). However, previous evidence regarding the efficacy of education on self-care and changing health behaviors in diabetic patients has resulted contradictory conclusions (13, 14). A study in Iran showed that education focusing on selfcare and changing health behavior was not effective in more than 50% of patients with diabetes (15). Also, the results of a systematic review indicated that educational programs are effective in diabetic patients in short term (≤9 months) (16).

It is believed that for diabetes self-care education in older adult patients, paying attention to their individual differences is crucial (17). It has been revealed that people learn differently in different situations because every person has a different learning style (18). The manner in which information is received and processed depends on the individual's learning style. Matching teaching strategies to individual learning styles can increase learner engagement, improve information recall, and allow for a more effective patient education session. Therefore, it seems that assessing the learning style of patients and then focusing teaching strategies to meet those individual styles can be very effective in self-care education and changing the health behavior of patients (19, 20). Learning styles can be considered as a method that people use to achieve a change by receiving new information and experiences in their minds and organizing and processing them (21). Learning styles usually changes with increasing age and changes in the environment. There are different methods to determine the learning style of individuals. One of the most important learning styles is based on Kolb learning style theory. Kolb believes that humans learn through four stages of feeling (doing assignments at home), watching (observation of PowerPoint and multimedia in the classroom), thinking (during teacher's teaching), and doing (group assignments and discussions). Based on this, Kolb defines four distinct learning styles in his learning styles theory as accommodating, diverging, converging and assimilating (18, 21, 22). The results of previous studies suggest that using individuated learning styles and adaptation of teacher's teaching method to learning style of individuals is a significant step towards better presentation of content and better understanding of it by learner. Hence, there is a need for more detailed studies to consider patients' learning styles for their education (20). There are very limited studies to evaluate the learning styles of older adult patients (23–25). Also, to the best of our knowledge there is no published study to evaluate the effect of self-care education based on Kolb learning style in older adult diabetic patients. Therefore, due to the importance of self-care practice and changing health behavior in the older people with diabetes, the aim of this study was to evaluate the effect of education based on Kolb's learning style on self-care behaviors of older adult patients with type II diabetes.

## Methods

**Study design and sample**: This randomized clinical trial was conducted on 62 older adult patients with type II diabetes referred to the Diabetes Center of Imam Reza Hospital affiliated to Bojnourd University of Medical Sciences, North Khorasan province, Iran. Data were collected from May to November 2020. Participants were selected using purposive sampling, according to the inclusion/exclusion criteria.

**Inclusion and exclusion criteria**: Inclusion criteria were age over 60 years, ability to read and write, at least three months of diagnosed diabetes, not attending formal diabetes educational sessions, no vision/hearing/neurological problems, fasting blood sugar between 140 to 400, HbA1C between 7 to 9%, and lack of experiencing acute and severe stress in the last six months. Exclusion criteria were needing to hospitalization during study period, experiencing acute/severe stress during the study, and absence from one of the education sessions.

**Questionnaires and data collection:** Data were collected using a three-part questionnaire including demographic characteristics, Kolb learning style questionnaire, and Toobert and Glasgow diabetes self-care activity questionnaire. Demographic characteristics questionnaire included patient's age, gender, body mass index (BMI), marital status, educational level, monthly income status, duration of diagnosis, and diabetes medications.

**Kolb learning style questionnaire:** The Kolb learning style questionnaire was used to assess patients' learning style. This questionnaire assesses individuals in two dimensions of objective experience-abstract conceptualization and reflective observation-active experimentation, and with combination of these dimensions, four main learning styles of convergent, divergent, assimilative and accommodative are obtained. This questionnaire consists of 12 items and four options are suggested for each item, and individuals can rank the options based on the explanation that each option provides for their performance. By adding the scores of each of these 4 options in 12 items of the questionnaire, four points are obtained. By subtracting the score of thinking method from the score of emotional method and by subtracting the score of the method of doing from the method of watching, two scores are obtained. These two scores are placed on two coordinate axes. These two axes make up four quadrants of a square, each quarter of which represents one of the learning styles. The validity and reliability of this questionnaire were confirmed by Simelane-Mnisi et al. (26). In the present study, the reliability of this tool was confirmed with Cronbach's alpha of 0.73.

**Toobert and Glasgow self-care questionnaire**: This questionnaire contains of 15 item to assess the frequencies of six specific self-management activities (5 items for diet, 2 items for exercise, 2 items for blood sugar testing, 4 items for foot care, 1 item for medications and 1 item for smoking) during the past week. Respondents mark the numbers of days (0–7) on which the indicated behaviors were performed. All scale scores range from 0 to 7 (exempt for smoking which was scored as 0 and 1), with higher scores suggesting better self-management, however, some items were scored inversely. The total score is obtained by adding up the scores of each item, which varies from 0 to 99 (27). The validity and reliability of this questionnaire were confirmed by Borhaninejad et al. with Cronbach's alpha of 0.77 (28). In the present study, the reliability of this tool was confirmed with Cronbach's alpha of 0.82.

**Intervention**: Older adult diabetic patients who met the inclusion criteria were randomly divided into two groups (intervention and control), using a computer-generated random-numbers table. Then, the researcher completed the Kolb learning style questionnaire for patients in the intervention group and the Toobert and Glasgow self-care questionnaire for both groups.

The educational intervention was conducted in the intervention and control groups in four sessions of 45 minutes (two sessions per week, in diabetes center of the hospital). The educational content was including the definition of diabetes, its causes, acute and chronic complications of diabetes, the way of adjusting the diet to the current family situation, adjusting the need for having physical activity and level of exercise according to the physical limitations of older people, prevention of complications during exercise, the importance of taking drugs, the way of measuring blood glucose with a glucometer, method of injecting insulin, and foot care. Participants in the intervention group were divided into four groups and received the educations based on their learning style. Patients with divergent learning style received their education using brainstorming and group discussion. Patients with convergent learning style received their education by showing diagrams and instructors' handwritten. Lectures and reading tutorials and having enough time to think were the teaching method for patients with assimilative learning style. For patients with accommodative learning style, role playing and computerized simulation were used as teaching method. For all patients in the control group, the same educational content was presented with a lecture and a booklet. Participants completed the questionnaires before and one month after the intervention.

**Ethical consideration:** The present study was registered in the Iranian Registry of Clinical Trials Database (IRCT20191109045365N1). The researchers visited the hospital after the study approval by the institutional ethics committee. The senior researcher explained the objectives of the study to the participants and invited them to participate in the study. Informed consent was obtained from the participants.

**Sample size calculation**: A total sample size of 70 patients was calculated with a confidence level of 90%, and power of 80% using the “formula for sample size calculation for comparison between two groups”. Considering that patients in the intervention group should be divided into 4 groups based on four types of learning styles, 40 patients were assigned to sub-groups (10 patients to each dominant learning style) and 30 patients for the control group.

**Statistical analysis:** Data were analyzed using the statistical package for the social sciences (SPSS) software (version 16.0, SPSS Inc., Chicago, IL, USA). Continuous variables were presented via mean (standard deviation) and number (percentage). Chi-square test was used to assess study variables in intervention and control groups. Mann-Whitney U test was also used to assess quantitatively abnormal variables such as age, diagnosis duration, and income status. Chi-square test was used to evaluate quantitatively normal variables such as sex, marital status, educational level, BMI, job status, and treatment method. The significance level was considered P <0.05.

**Data sharing:** All relevant data and methodological detail pertaining to this study are available to any interested researchers upon reasonable request to corresponding author.

## Results

**Figure 1:**
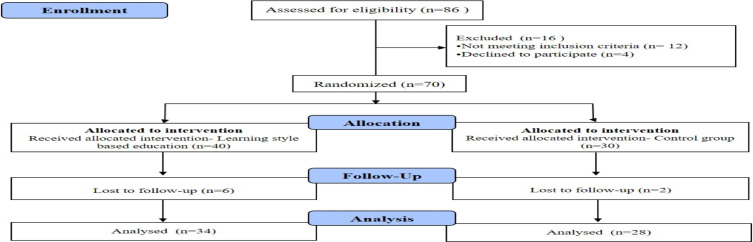
Flowchart of the study

**Participants**: From 70 patients who were allocated into the two groups, 8 patient's losses to follow-up during sturdy period. In total, 62 older adults with type II diabetes completed the present study and data from all these patients were analyzed (Figure 1). The mean age of participants was 66.42 (SD=5.27) years. Of the participants, 59.68% were female, 66.13% were married, and 54.84% were on oral diabetes medications. There was no statistically significant difference between two groups in terms of demographic characteristics. The demographic characteristics of the participants were presented in Table 1.

**Table 1:** Demographic characteristics of participants

Variables	Groups					
	
	Intervention (n=34)				Control (n=28)	*P-value*

	Convergent (n=9)	Divergent (n=8)	Assimilator (n=9)	Accommodator (n=8)		
**Age (y)**	65.90 (SD=5.50)	65.50 (SD=5.30)	68.20 (SD=4.60)	67.00 (SD=5.10)	66.00 (SD=5.50)	0.780*
**Sex**						
**Male**	3 (33.33)	5 (62.50)	4 (44.44)	4 (50.00)	9 (32.14)	0.258**
**Female**	6 (66.67)	3 (37.50)	5 (55.56)	4 (50.00)	19 (67.86)	
**Marital status**						
**Single**	3 (33.33)	1 (12.50)	6 (66.67)	2 (25.00)	9 (32.14)	0.529**
**Married**	6 (66.67)	7 (87.50)	3 (33.33)	6 (75.00)	19 (67.86)	
**Level of Education**						
**Cycle**	4 (44.45)	2 (25.00)	7 (77.78)	4 (50.00)	18 (64.29)	
**Under diploma**	2 (22.22)	1 (12.50)	1 (11.11)	2 (25.00)	3 (10.71)	0.368**
**Diploma and** **higher**	3 (33.33)	5 (62.50)	1 (11.11)	2 (25.00)	7 (25.00)	
**BMI**	28.82 (SD=4.19)	27.13 (SD=5.13)	29.44 (SD=5.99)	29.36 (SD=5.3)	27.27 (SD=9.41)	0.713**
**Job status**						
**Unemployed**	2 (22.22)	0 (00)	0 (00)	1 (12.50)	2 (7.14)	
**Retired**	2 (22.22)	1 (12.50)	0 (00)	0 (00)	2 (7.14)	
**Employed**	3 (33.34)	4 (50.00)	1 (11.12)	3 (37.50)	7 (25.00)	0.097**
**Housewives**	2 (22.22)	2 (25.00)	4 (44.44)	4 (50.00)	15 (53.58)	
**Others**	0 (00)	1 (12.50)	4 (44.44)	0 (00)	2 (7.14)	
**Income status**						
**Low**	1 (11.11)	1 (12.50)	1 (11.11)	4 (50.00)	12 (42.86)	
**Moderate**	5 (55.56)	7 (87.50)	6 (66.67)	3 (37.50)	15 (53.57)	0.061*
**Higher**	3 (33.33)	0 (00)	2 (22.22)	1 (12.50)	1 (3.57)	
**Diagnosis duration**	97.78 (SD=17.92)	81.50 (SD=13.30)	76.33 (SD=11.05)	77.88 (SD=21.67)	78.29 (SD=11.77)	0.948*
**Treatment method**						
**Oral**	7 (77.78)	5 (62.50)	5 (55.56)	4 (50.00)	13 (46.43)	0.520**
**Injection insulin**	0 (00)	1 (12.50)	2 (11.12)	2 (25.00)	4 (14.28)	
**Both methods**	2 (22.22)	2 (25.00)	2 (11.12)	2 (25.00)	11 (39.29)	

**SD**: Standard Deviation. BMI: Body Mass Index; Data are presented as number (percentage) and mean (standard deviation)

* p-value was obtained with Mann-Whitney U test

** p-value was obtained with Chi-square test.

**Outcomes:** As shown in Table 2, the mean self-care scores of the older adult patients with type II diabetes in the intervention and control groups were 58.15 (SD=10.71) and 44.7 (SD=12.12), respectively (P<0.001). Patients with accommodating learning style, who received their style-based education, had more improvement in their self-care behaviors scores (P<0.001). Also, the educational intervention had a significant effect on self-care dimensions including diet and foot care (P<0.001). But its effect on the area of exercise (P=0.499) and blood sugar testing (P= 0.057) and medications (P=0.797) were non-significant.

**Table 2:** Self-care dimensions of participants

Dimensions of self-care	Groups					
	
	Intervention (n=34)				Control (n=28)	*P-value*

	Convergent (n=9)	Divergent (n=8)	Assimilator (n=9)	Accommodator (n=8)		
**Foot care**						
**Before** **intervention**	6.55 (SD=5.93)	3.12 (SD=3.35)	4.00 (SD=1.87)	2.87 (SD=1.40)	6.67 (SD=6.28)	0.217
**After** **intervention**	16.10 (SD=4.37)	13.10 (SD=6.31)	13.20 (SD=5.33)	20.75 (SD=4.26)	9.80 (SD=3.30)	<0.001

**Blood sugar** **testing**						
**Before** **intervention**	7.00 (SD=4.71)	6.87 (SD=4.29)	3.33 (SD=3.57)	7.75 (SD=3.69)	4.17 (SD=4.36)	0.072
**After** **intervention**	9.30 (SD=0.70)	9.10 (SD=0.35)	7.88 (SD=2.20)	9.82 (SD=0.35)	6.75 (SD=3.94)	0.057

**Exercise**						
**Before** **intervention**	3.33 (SD=3.93)	5.12 (SD=6.03)	3.44 (SD=2.65)	4.00 (SD=4.17)	3.02 (SD=2.57)	0.686
**After** **intervention**	4.00 (SD=1.10)	5.80 (SD=1.90)	4.30 (SD=1.40)	3.60 (SD=0.83)	3.40 (SD=0.48)	0.499

**Diet**						
**Before** **intervention**	19.88 (SD=7.94)	20.75 (SD=4.30)	14.00 (SD=4.47)	15.50 (SD=3.80)	16.00 (SD=4.43)	0.035
**After** **intervention**	20.88 (SD=2.30)	24.50 (SD=3.30)	21.27 (SD=2.80)	18.44 (SD=4.36)	17.57 (SD=3.80)	<0.001

**Medications**						
**Before** **intervention**	6.56 (SD=1.33)	5.25 (SD=3.24)	5.00 (SD=3.04)	5.60 (SD=2.66)	5.29 (SD=2.73)	0.761
**After** **intervention**	6.50 (SD=1.33)	7.00 (SD=0)	6.66 (SD=1.00)	7.00 (SD=0)	6.53 (SD=1.47)	0.797

**Total**						
**Before** **intervention**	44.10 (SD=10.50)	42.00 (SD=9.40)	36.20 (SD=13.00)	30.20 (SD=10.5)	35.70 (SD=13.00)	0.122
**After** **intervention**	58.40 (SD=8.30)	60.50 (SD=10.70)	50.40 (SD=12.20)	63.20 (SD=3.40)	44.70 (SD=12.70)	<0.001

**SD**: Standard Deviation, Data are presented as mean (standard deviation), P-value was obtained with Chi-square test.

## Discussion

The results of the present study showed that the education based on Kolb's learning style had a significant effect on improving self-care in the older adult with type II diabetes. On the other hand, the most effective of Kolb's learning style-based education on improving self-care in older people with type II diabetes was in patients with accommodating learning style. Also, the educational intervention had a significant effect on diabetes self-care dimensions including diet and foot care. In line with the results of the present study, the results of a study by Saleh Moghadam et al. in using the VARK learning style in educating diabetic patients, showed a significant improvement in fasting blood glucose and glycosylated hemoglobin levels in diabetic patients (29). The tool used in the mentioned study was the VARK learning style which was different from that of our study, but it indicated the positive efficacy of using individualized learning style on patient education. Also, Rashidi et al. showed that teaching based on Kolb's learning style increased the university students' creativity and academic achievement (30). However, it is believed that determining the learning style of people merely by filling out a questionnaire is not possible and it is a very complex task and teachers' perception is effective in determining of it (31).

In line with the results of present study, in a study by Janet et al. with aim to investigate the preference of Kolb learning style among older adults showed that with increasing age, the tendency to use accommodating learning style increases in the older people (25). Also, in a study conducted by Biabani et al. with aim to evaluate the relationship between Kolb learning styles and gender with the level of English language learning by Iranian students, the results showed that these students were pragmatic and learn better by using accommodating learning style (32). Also, the results of the present study revealed that elderly education based on Kolb learning styles did not have the same effect on self-care in older adult with type II diabetes in all areas, and this might indicate that self-care is affected by other factors other than people learning. Also, the results of our study showed that the effect of Kolb's learning styles on selfcare in older people with type II diabetes was significant in the dimensions of diet and foot care. In line with the results of our study, the results of a study by Shin et al. revealed that with increasing health literacy in the older people, their abilities in the areas of diet control and physical activity increase (33). Inconsistent with the results of the present study, results of a study by White et al. showed that a 4-week educational program to promote healthy nutrition in adults with type II diabetes and heart disease did not have a significant effect (34). However, it has been previously confirmed that an insufficient self-care information is the most important barrier for self-care management and increasing health literacy is the best solution to increase self-care in the older people (35, 36). Moreover, the results of our study revealed that the effect of Kolb's learning styles on self-care in the older adult with type II diabetes was not significant in the areas of physical activity, blood glucose control, and medications. In line with the results of our study, the results of a study by Kim et al. showed that the older people have a lower tendency to participate in sports activities due to the presence of chronic diseases (37). Inconsistent with the results of our study, some previous studies showed an increase in physical activity in the older people after providing education (33, 38). Also, in line with the results of our study in the area of adherence to the medications, it has been indicated that adherence to the medications increases with advancing age (39). In addition, in line with those of our study, some studies showed that the area of blood glucose control did not change after patients' education (35, 40), however, these results was not confirmed by other studies (41). Not evaluating other confounding factors such as economic and cultural factors which can possibly affect the self-care behaviors of older adult diabetic patients is one of the limitation of this study. Also, the present study was conducted only in one center, which can limit the generalizability of the study results.

In conclusion, according to the results of present study it seems that self-care education based on Kolb's learning style can improve the self-care ability of older people with type II diabetes. Therefore, it is suggested to use Kolb's learning style in the self-care educational process of older adult with type II diabetes to improve their self-care practice.
